# Immunomodulatory Effects of the Calcitriol Analog Paricalcitol

**DOI:** 10.3390/biom16101413

**Published:** 2026-09-29

**Authors:** Dionysios N. Vrachnis, Maria Apostolopoulou, Rodis D. Paparodis, Eleni Dermitzaki, George Mastorakos, Ioanna N. Grivea, Alexandra Bargiota, Dimitrios T. Papadimitriou

**Affiliations:** 1 Department of Endocrinology and Metabolic Diseases, Larissa University Hospital, 41110 Larissa, Greece; maria-apostolopoulou@hotmail.com (M.A.);; 2 Medical School, National and Kapodistrian University of Athens, 11527 Athens, Greece; 3 Faculty of Medicine, School of Health Sciences, University of Thessaly, 41110 Larisa, Greece; 4 Hellenic Endocrine Network, 10563 Athens, Greece; rodis@paparodis.gr; 5 Endocrinology, Diabetes and Metabolism Clinics, 26221 Patras, Greece; 6 Division of Endocrinology, Diabetes and Metabolism, Department of Medicine, Loyola University Chicago, Chicago, IL 60153, USA; 7 Department of Medicine, Edward Hines Jr. VA Hospital, Hines, IL 60141, USA; 8 Pediatric Endocrine Clinics, 15125 Athens, Greece; 9 Unit of Endocrinology, Diabetes Mellitus and Metabolism, ARETAIEION University Hospital, School of Medicine, National and Kapodistrian University of Athens, 11528 Athens, Greece; 10 Department of Pediatrics, University of Thessaly, 41110 Larissa, Greece

**Keywords:** paricalcitol, vitamin D receptor, immunomodulation, inflammation, autoimmune diseases, chronic kidney disease, regulatory T cells, type 1 diabetes

## Abstract

Paricalcitol (19-nor-1,25-dihydroxyvitamin D_2_) is a synthetic vitamin D receptor (VDR) agonist used for the treatment of secondary hyperparathyroidism mainly in chronic kidney disease (CKD), with a 10-times lower calcemic potential and 3-times less potent to the VDR than calcitriol. This narrative review examines the available preclinical and clinical evidence on the immunomodulatory actions of paricalcitol beyond mineral metabolism. Experimental studies show that paricalcitol can modulate immune-cell function, inflammatory signaling, oxidative stress and fibrosis through VDR-dependent pathways. Human evidence is derived mainly from patients with CKD and kidney transplantation, where reductions in inflammatory and oxidative stress markers have been reported; however, findings are not consistent across all studies. Evidence in other immune-mediated conditions, including IgA nephropathy, psoriasis, atopic dermatitis, autoimmune alopecia, and type 1 diabetes (T1D), remains limited and is based largely on case series and reports and non-RCTs. The PRECAL study (ISRCTN17354692) provides preliminary evidence that timely VDR activation may influence the course of T1D, requiring controlled confirmation. No dosing regimen has yet been established for immunomodulatory use, while the calcitriol replacement daily dose × 3/day (given the relevant potency) has been proven reasonably safe and effective in the PRECAL study. Predictive biomarkers of response are lacking, warranting further basic research.

## 1. Introduction

Vitamin D is traditionally recognized as a key regulator of calcium and phosphate homeostasis and plays an essential role in bone health. However, over the last two decades, growing evidence has demonstrated that vitamin D also exerts important effects on the immune system. The vitamin D receptor (VDR), a ligand-activated nuclear receptor, is expressed in a wide range of immune cells, including monocytes, macrophages, dendritic cells (DC), and T and B lymphocytes [1,2]. Through regulation of gene transcription, VDR signaling modulates antigen presentation, cytokine production, immune-cell differentiation, and the maintenance of immune tolerance. Growing evidence indicates that immune dysregulation contributes not only to classical autoimmune diseases but also to chronic inflammatory disorders including chronic kidney disease (CKD) and diabetes mellitus. This has renewed interest in vitamin D receptor agonists as potential immunomodulatory therapies.

Paricalcitol (19-nor-1,25-dihydroxyvitamin D_2_) is a synthetic vitamin D analogue developed to selectively activate the VDR while producing substantially lower calcemic effects than calcitriol. Compared with calcitriol, paricalcitol exerts significantly less stimulation of intestinal calcium absorption while maintaining effective suppression of parathyroid hormone secretion [3]. Since its approval by the U.S. Food and Drug Administration in 1998 for the prevention and treatment of secondary hyperparathyroidism (SHPT) in patients with CKD, paricalcitol has been widely used in clinical practice and has demonstrated a favorable safety profile [4,5]. More recently, accumulating experimental and clinical evidence has suggested that paricalcitol may exert important immunomodulatory actions, raising interest in its potential application beyond SHPT and CKD.

The 19-nor structure of paricalcitol, characterized by the absence of the carbon-19 methylene group, is associated with reduced calcemic activity while preserving VDR-mediated transcriptional activity. Importantly, the biological activity of vitamin D analogues depends not only on their binding affinity for the VDR but also on how they interact with the receptor. Different VDR ligands can induce slightly different receptor conformations, including changes in the positioning of helix 12, which may influence the recruitment of transcriptional co-regulators and consequently the expression of VDR-regulated genes. This may help explain why paricalcitol can produce different biological responses from calcitriol in different tissues and cell types. However, the precise mechanisms responsible for the tissue selectivity of paricalcitol remain incompletely understood [3,6].

**Figure 1:**
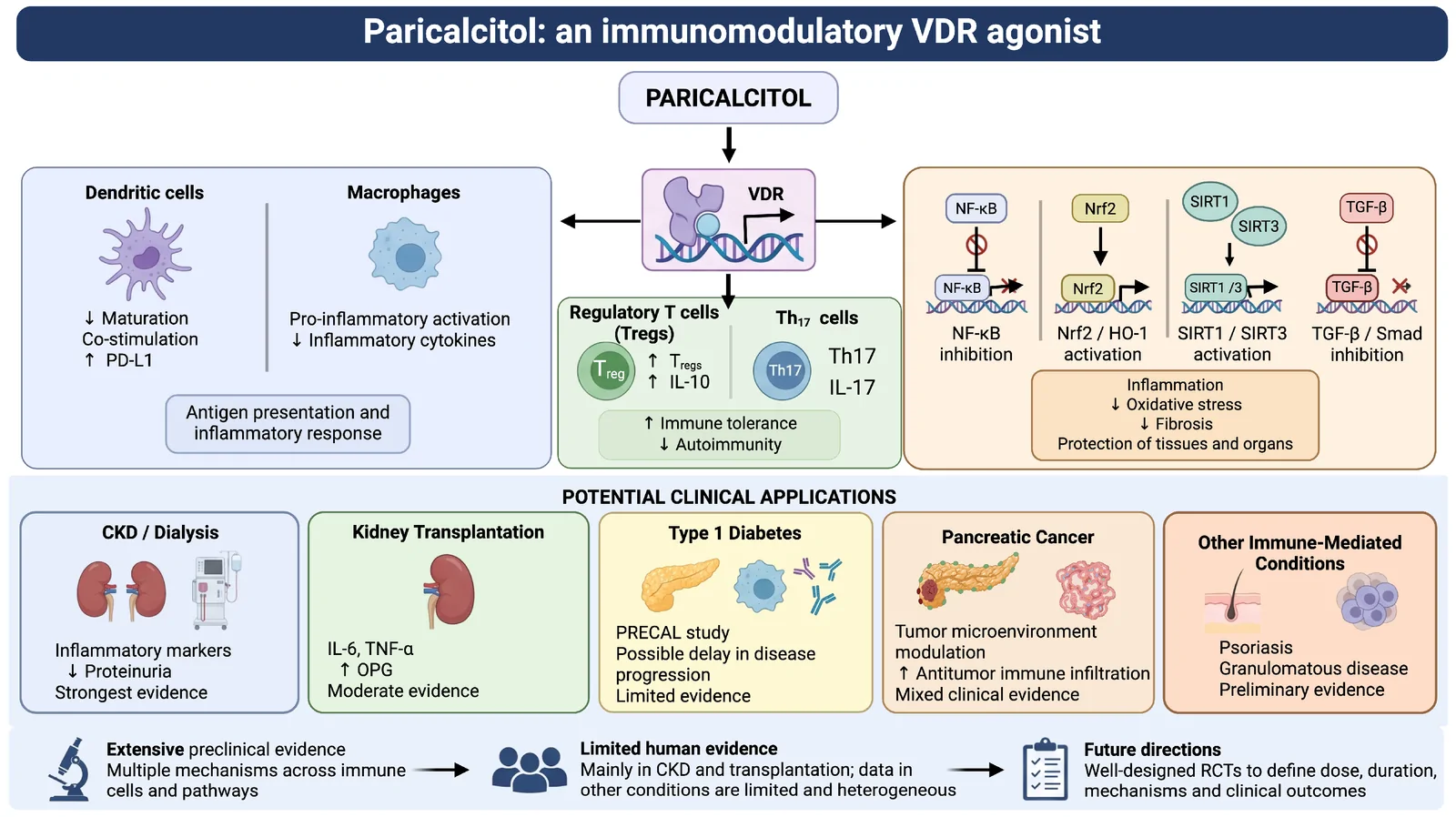
Immunomodulatory mechanisms and potential clinical applications of paricalcitol. Paricalcitol-mediated VDR activation modulates immune-cell function and intracellular signaling, contributing to reduced inflammation, oxidative stress and fibrosis, and enhanced immune tolerance. The figure also summarizes the current clinical and preclinical evidence across different disease settings. Abbreviations: CKD, chronic kidney disease; HO-1, heme oxygenase-1; IL, interleukin; NF-κB, nuclear factor kappa B; Nrf2, nuclear factor erythroid 2-related factor 2; OPG, osteoprotegerin; PD-L1, programmed death-ligand 1; RCTs, randomized controlled trials; SIRT1/3, sirtuin 1/sirtuin 3; TGF-β, transforming growth factor beta; Th17, T helper 17 cells; Tregs, regulatory T cells; VDR, vitamin D receptor; ↑, increase or upregulation; ↓, decrease or downregulation.

To date, the immunomodulatory properties of paricalcitol as a distinct pharmacological entity have not been systematically addressed. In this narrative review, we discuss the available preclinical and clinical evidence on the immunomodulatory properties of paricalcitol, focus on the underlying cellular and molecular mechanisms, summarize ongoing clinical trials, and discuss the safety profile, potential applications beyond its licensed indication for SHPT, as well as current limitations and future perspectives. The main immunomodulatory mechanisms and potential clinical applications of paricalcitol are summarized in Figure 1.

## 2. Mechanistic Studies and Preclinical Evidence

Dendritic Cells (DCs) are specialized antigen-presenting cells that serve as key regulators of immune responses [7]. Derived primarily from bone marrow progenitors and, under certain inflammatory conditions, from circulating monocytes, DCs continuously survey peripheral tissues for foreign and self-antigens. Upon activation, they process and present antigens to T lymphocytes while providing the co-stimulatory signals required for effective adaptive immune responses. Beyond initiating immunity, DCs play a fundamental role in maintaining immune homeostasis by balancing inflammatory responses with mechanisms of peripheral tolerance [8].

DCs respond to paricalcitol through VDR activation. In both human and animal studies, vitamin D promotes antigen uptake while inhibiting dendritic-cell maturation, resulting in a reduced ability to activate antigen-specific CD8^+^ T cells. In an in vitro study, Sochorová et al. used human monocyte-derived DCs and compared three experimental conditions: untreated control cells, calcitriol-treated cells and paricalcitol-treated cells. Compared with controls, both calcitriol and paricalcitol inhibited DC maturation, as evidenced by lower expression of CD80, CD83, CD86 and MHC molecules. In addition, both agents reduced IL-12 production and decreased the expansion of antigen-specific T cells. Upregulation of the inhibitory checkpoint molecule PD-L1 was also observed in both treatment groups. Thus, the immunomodulatory effects of paricalcitol were comparable to those of calcitriol, supporting its ability to modulate immune responses through VDR activation [9]. Furthermore, upregulation of PD-L1 by paricalcitol has been observed in other experimental contexts [9,10,11]. In an experimental rat model of granulomatous inflammation, early administration of paricalcitol significantly reduced the number of immature granulomas and increased mature granuloma formation compared with untreated animals [11]. These histological changes were accompanied by a significant reduction in CD68-positive macrophages and CD3-positive T cells, a significant increase in CD1a-positive cells when treatment was initiated at sensitization, and a marked, significant upregulation of PD-L1 expression. In contrast, delayed treatment produced no significant histological or immunological changes.

In addition, VDR activation increases the number of regulatory T cells (Tregs), which suppress IL-17 production, and reduces the secretion of pro-inflammatory cytokines such as TNF-α and IL-8. In a murine model of peritoneal fibrosis, mice were allocated to three groups: a control group, a group exposed to peritoneal dialysis fluid (PDF), and a group exposed to PDF plus paricalcitol. Compared with mice receiving PDF alone, paricalcitol increased the frequency and absolute number of FoxP3^+^ regulatory T cells, particularly CD8^+^ Tregs expressing CTLA-4 and TGF-β. Furthermore, regulatory T cells isolated from the paricalcitol group suppressed IL-17 production, suggesting that VDR activation promotes an immunoregulatory environment that may contribute to reduced inflammation and fibrosis [12]. Eleftheriadis et al., in an in vitro study using human peripheral blood mononuclear cells, demonstrated that paricalcitol significantly reduced TNF-α and IL-8 production under both basal and pro-inflammatory conditions [13].

Much of the available mechanistic evidence is derived from experimental models of renal disease, reflecting the established use of paricalcitol in CKD. However, the pathways identified may also be relevant to inflammation in other tissues and disease settings. Several studies suggest that the immunomodulatory effects of VDR agonists are mediated, at least in part, through inhibition of the NF-κB signaling pathway. In addition to its effects on DCs, paricalcitol has been shown to suppress inflammation through NF-κB inhibition in other experimental settings. In a murine model of obstructive nephropathy, paricalcitol reduced renal infiltration by T lymphocytes and macrophages and decreased the expression of pro-inflammatory cytokines and chemokines. Interestingly, this effect was mediated by the formation of a VDR–NF-κB complex that prevented NF-κB from binding to the promoters of inflammatory genes, representing a mechanism distinct from classical NF-κB inhibition [14]. Dáňová et al. generated tolerogenic DCs from human monocytes using a combination of paricalcitol and dexamethasone and compared them with conventionally generated DCs. The tolerogenic DCs showed reduced expression of co-stimulatory molecules, increased ILT-3 and PD-L1 expression, enhanced IL-10 production and reduced T-cell stimulatory capacity, even after exposure to pro-inflammatory conditions. These changes were accompanied by reduced NF-κB, suggesting that suppression of NF-κB signaling contributed to the maintenance of the tolerogenic phenotype. However, because paricalcitol was administered together with dexamethasone, the individual contribution of paricalcitol cannot be fully distinguished, highlighting the need for studies using paricalcitol alone [10].

Paricalcitol has also been shown to exert cytoprotective effects through activation of the Nrf2/HO-1 pathway. In a murine model of cisplatin-induced acute kidney injury, paricalcitol promoted nuclear translocation of Nrf2, upregulated HO-1 expression, and reduced oxidative stress markers, resulting in decreased inflammatory cell infiltration and improved renal function [15].

Another mechanism that has been described is the suppression of the renin–angiotensin–aldosterone system (RAAS) by paricalcitol through downregulation of renin gene expression [16]. In a murine model of chronic renal failure, treatment with paricalcitol reduced angiotensinogen, renin, renin receptor and VEGF mRNA levels by 30–50%.

Paricalcitol has also been associated with anti-fibrotic effects. In an adriamycin-induced nephropathy model, treatment with paricalcitol reduced TGF-β1/Smad2/3 activity and lowered the expression of mesenchymal markers in the kidney, suggesting a potential role in limiting endothelial-to-mesenchymal transition and the development of renal fibrosis, along with improved renal function [17]. In addition, paricalcitol reduced inflammatory cytokine production, decreased macrophage infiltration and attenuated MAPK pathway activation [18].

VDR is expressed in hepatic cells. Malladi et al. conducted a preclinical study using murine models of non-alcoholic fatty liver disease (NAFLD) together with complementary in vitro experiments in HepG2 hepatocytes. Animals were allocated to three groups: control, NAFLD and paricalcitol-treated NAFLD [19]. This study describes a potential epigenetic mechanism through which paricalcitol modulates inflammation and oxidative stress. Paricalcitol upregulated SIRT1 and SIRT3, two NAD+-dependent deacetylases, leading to reduced acetylation of both NF-κB and FOXO3a. Deacetylation of NF-κB suppressed the transcription of pro-inflammatory genes, whereas deacetylation of FOXO3a restored its transcriptional activity and promoted the expression of antioxidant enzymes, including superoxide dismutase and catalase. Accordingly, paricalcitol-treated animals exhibited lower IL-1β and TNF-α levels, reduced hepatic oxidative stress, improved liver function tests, and less severe histological features of NAFLD compared with untreated animals. However, this study was limited by its preclinical design, small sample sizes for several analyses, and has not yet been confirmed in human studies.

Paricalcitol has been associated with neuroprotective effects. In Alzheimer’s disease, reduced vitamin D status has been associated with increased risk of cognitive decline and neurodegeneration [20]. Wu et al. investigated the effects of paricalcitol in APP/PS1 mice, a commonly used model of Alzheimer’s disease [21]. Animals were assigned to either a paricalcitol-treated group or a control group and followed for 15 weeks. Compared with untreated animals, paricalcitol reduced iron accumulation in the cortex and hippocampus and lowered the expression of proteins involved in iron uptake. Since hyperphosphorylation of Tau is a key pathological feature of Alzheimer’s disease, it is noteworthy that paricalcitol was also associated with reduced Tau phosphorylation and lower GSK3β activity, suggesting a potential role in slowing disease-related neurodegenerative changes. Moreover, in a prospective randomized rat model of transient global cerebral ischemia, animals were assigned to either paricalcitol or saline treatment [22]. Paricalcitol significantly reduced neuronal degeneration in the hippocampal CA1 region and improved early neurological recovery. However, no significant differences were observed in motor coordination, memory function or survival.

Beyond the nervous system, anti-inflammatory effects of paricalcitol have also been observed in cardiovascular models. In a murine model of lipopolysaccharide (LPS)-induced endotoxemia, animals were assigned to control, paricalcitol, LPS and LPS plus paricalcitol groups. Decreased activation of NF-κB has been observed, which subsequently reduced the expression of Intercellular Adhesion Molecule-1 (ICAM-1), Vascular Cell Adhesion Molecule-1 (VCAM-1), fractalkine and TNF-α, both in endothelial cells and myocardial tissue in the paricalcitol group. In addition, paricalcitol improved myocardial microvascular permeability, suggesting a protective effect on endothelial function during systemic inflammation [23]. In several animal models, paricalcitol was associated with reduced cardiac hypertrophy, preserved ejection fraction and lower levels of inflammation and oxidative stress [24,25,26].

Similar anti-inflammatory and antioxidant effects have been described in experimental diabetes. Paricalcitol was also investigated in a streptozotocin/high-fat diet-induced rat model of type 2 diabetes (T2D) [27]. Rats received intraperitoneal paricalcitol (0.8 μg/kg/day) for 12 weeks. Compared with untreated diabetic animals, paricalcitol significantly reduced pancreatic TNF-α and IL-1β levels and significantly increased IL-2, adiponectin and antioxidant enzyme activity. Significant improvements were also observed in glycemic parameters, including fasting and postprandial blood glucose, fasting insulin, C-peptide and HOMA-IR, together with preservation of pancreatic islet morphology.

Overall, experimental studies consistently indicate that paricalcitol exerts anti-inflammatory, antioxidant, immunomodulatory and anti-fibrotic effects through VDR-dependent mechanisms. Table 1 summarizes the immunomodulatory mechanism of paricalcitol. These findings provide a biological rationale for its potential therapeutic application beyond mineral metabolism, although confirmation in human studies remains limited.

## 3. Evidence from Human Studies

The preclinical evidence provides a biological rationale for investigating the immunomodulatory effects of paricalcitol in humans. Clinical studies have evaluated paricalcitol across a range of immune-mediated and inflammatory conditions, although the evidence remains limited and heterogeneous. Most of the available clinical evidence comes from patients with CKD and end-stage kidney disease, where chronic low-grade inflammation is common and associated with adverse clinical outcomes. Early clinical studies provided some evidence of an anti-inflammatory effect. In a prospective in vivo study, 40 patients with stage 4–5 CKD and SHPT, together with 20 healthy controls, were included. Patients received a single intravenous bolus of paricalcitol (5 μg), and inflammatory markers were measured at baseline and 24 h after treatment [28]. A single dose significantly reduced circulating NGAL, a marker of tubular injury and systemic inflammation, as well as IL-17, IL-6, IL-1β, TNF-α, IFN-γ and erythrocyte sedimentation rate (ESR), whereas changes in high-sensitivity C-reactive protein (hsCRP), albuminuria, proteinuria and parathyroid hormone (PTH) did not reach statistical significance. Similarly, in a prospective single-arm study [29], which enrolled 19 patients with end-stage CKD undergoing maintenance hemodialysis and SHPT, 12 weeks of intravenous paricalcitol was associated with reductions in CRP, TNF-α, IL-6 and IL-18, and increased IL-10 levels. Markers of oxidative stress also decreased, while antioxidant defenses were significantly improved.

However, these findings have not been consistently reproduced in randomized trials. In a randomized, double-blind, placebo-controlled pilot trial of 24 patients with stage 2–3 CKD, paricalcitol at 1 or 2 μg/day reduced albuminuria, while hsCRP decreased only with the higher dose [30]. No significant effects were observed on endothelial function, ambulatory blood pressure, GFR or PTH. In another RCT, which involved 36 patients with stage 3–4 CKD, treatment with paricalcitol 2 μg daily was associated with better preservation of endothelial function, whereas blood pressure, pulse wave velocity, albuminuria and CRP remained unchanged [31]. Similarly, in a crossover RCT of 26 patients with stage 3–4 CKD and albuminuria, paricalcitol (2 μg/day) for 6 weeks did not significantly affect renin, angiotensin II, aldosterone or 24-h albuminuria [32]. Finally, in an RCT with 60 patients with stage 3b-5 CKD and anemia, paricalcitol (1 μg/day) led to increased hemoglobin and reduced proteinuria compared with calcitriol; however, hsCRP levels remained unchanged [33].

The immunomodulatory effects of paricalcitol have also been investigated in patients undergoing kidney transplantation. In an open-label RCT of 74 de novo renal transplant recipients, paricalcitol increased circulating osteoprotegerin (OPG) and TNFRSF11B expression in renal graft biopsies, but did not significantly affect the other 13 biomarkers of inflammation, endothelial activation and fibrosis, which were examined in this study [34]. In contrast, a prospective study of 31 kidney transplant recipients with SHPT reported reductions in hsCRP, IL-6 and TNF-α levels as well as lower TNF-α/IL-10 and IL-6/IL-10 ratios after 3 months of treatment [35]. These findings were supported by a larger RCT, which included 168 kidney transplant recipients with persistent proteinuria, in which paricalcitol reduced circulating IL-6 and TGF-β levels, although plasma renin activity and aldosterone remained unchanged [36]. Collectively, the transplant literature also suggests a possible anti-inflammatory effect; however, the consistency and intensity of this effect vary across the studies.

Evidence in immune-mediated renal disease is more limited. In a prospective study of seven patients with refractory IgA nephropathy, paricalcitol (1 μg/day) was administered for at least 6 months, followed by the addition of subcutaneous secukinumab, an IL-17A inhibitor [37]. Sequential treatment was associated with a 71% reduction in proteinuria and stabilization of renal function, along with changes in circulating T-cell populations. However, because secukinumab was subsequently added after paricalcitol, the specific contribution of paricalcitol cannot be determined.

Outside renal disease, the clinical evidence becomes considerably sparser. In patients with type 2 diabetes (T2D) who are characterized by insulin resistance and chronic low-grade inflammation [38], an RCT of 60 patients with T2D and stage 3–4 CKD found no significant effect of paricalcitol (1 μg/day) on endothelial function, hsCRP, IL-6 or TNF-α [39]. The larger VITAL study, a multicentre RCT enrolling 281 patients with T2D and albuminuria receiving stable angiotensin-converting enzyme inhibitor or angiotensin receptor blocker therapy, evaluated paricalcitol (1 or 2 μg/day) versus placebo for 24 weeks but did not assess inflammatory markers, precluding conclusions regarding the immunomodulatory effects of paricalcitol in this population [40].

Evidence for the immunomodulatory effects of paricalcitol in dermatological disease remains limited to small studies and case reports. In a double-blind, self-controlled pilot study involving 11 patients with moderate plaque psoriasis, topical paricalcitol (15 μg/g) applied once daily for 12 weeks significantly reduced erythema, scaling and plaque thickness compared with placebo [41]. Isolated reports have also suggested potential effects in severe atopic dermatitis and alopecia areata. In a child with severe, treatment-refractory atopic dermatitis, clinical improvement achieved with calcitriol was maintained after switch to paricalcitol (2 μg three times daily) because of hypercalciuria [42]. Partial hair regrowth was also reported in a child with alopecia areata universalis treated with the same paricalcitol dose after calcitriol-induced hypercalcemia and hypercalciuria, although the response was limited and relapsed after treatment discontinuation [43].

Most recently, VDR activation was studied in ocular disease. A case report described a 57-year-old woman with recurrent HSV-1 keratitis and corneal perforation, who received calcifediol (20 μg/day) together with low-dose paricalcitol as adjunctive therapy following surgical treatment [44]. Corneal re-epithelialization and thickening were observed, without recurrence of HSV-1 keratitis or orolabial herpes during follow-up. However, as this was a single-patient case report and paricalcitol was administered in combination with calcifediol and standard therapy, its specific immunomodulatory contribution cannot be determined.

Overall, the available clinical evidence presents a less consistent picture than the experimental data. Anti-inflammatory effects are most apparent in CKD and kidney transplantation, although even in these settings they still need to be reproduced in randomized trials. Evidence outside renal disease remains preliminary and is largely derived from smaller studies or case reports. Whether the immunomodulatory properties of paricalcitol can translate into clinical relevance in autoimmune disease remains unknown and warrants further investigation, particularly in type 1 diabetes (T1D). The available clinical evidence is summarized in Table 2.

## 4. Paricalcitol and Type 1 Diabetes

Type 1 diabetes mellitus accounts for approximately 5–10% of all diabetes cases and is an autoimmune disorder characterized by progressive immune-mediated destruction of pancreatic β-cells, ultimately leading to absolute insulin deficiency and lifelong dependence on exogenous insulin [48]. Impaired VDR signaling, particularly during early life, has been associated with an increased risk of T1D. The autoimmune response in T1D is characterized by inflammatory infiltration of the pancreatic islets by macrophages and autoreactive T cells, while Th17 cells and impaired regulatory Treg function further contribute to β-cell destruction and loss of immune tolerance [49].

Experimental studies have shown that vitamin D and its analogues may protect pancreatic β-cells by reducing apoptosis and preserving insulin secretion. Proposed mechanisms include upregulation of the anti-apoptotic protein A20, modulation of Fas-mediated cell death, protection against IL-1β-induced cytotoxicity, and improved intracellular calcium handling [49]. Consistent with these findings, studies in non-obese diabetic (NOD) mice showed that vitamin D and its analogues reduced the incidence of T1D, preserved pancreatic β-cell mass and insulin content, and attenuated insulitis [50]. Additional studies reported improved glycemic control, enhanced insulin secretion, and prolonged islet graft survival when vitamin D analogues were combined with immunomodulatory therapies [50,51]. However, as most studies were performed in vivo, the relative contributions of direct β-cell protection and immune modulation could not be clearly distinguished.

As a VDR agonist, paricalcitol promotes a more tolerogenic immune profile by reducing dendritic cell antigen presentation, shifting macrophages toward an anti-inflammatory phenotype, enhancing Treg responses, and suppressing Th1 and Th17 differentiation. These effects are accompanied by increased production of anti-inflammatory cytokines and reduced expression of pro-inflammatory mediators. Its lower calcemic activity compared with calcitriol may be advantageous if prolonged VDR activation is required.

A small phase I study in nine patients with long-standing T1D also examined the effect of tolerogenic DCs generated ex vivo using calcitriol and dexamethasone [52]. The cells were loaded with a proinsulin peptide and administered intradermally in two doses. Treatment reduced proinsulin-specific T-cell autoreactivity, with the effect persisting for more than two years, and significantly increased a subset of activated Tregs. No evidence of generalized immune suppression was observed. Although paricalcitol was not used in this study, these findings provide clinical proof of concept that VDR-mediated modulation of DCs can promote antigen-specific immune tolerance in T1D.

The PRECAL (Secondary Prevention of Type 1 Diabetes with Oral Calcitriol and Analogs) study was a prospective, interventional clinical study evaluating whether calcitriol and paricalcitol could delay or prevent the progression of T1D in children at high risk of developing the disease [53]. A total of 42 children positive for one or more islet autoantibodies and/or carrying high-risk HLA haplotypes were included in the final analysis. Treatment was individualized according to age, disease stage and calcium metabolism. Calcitriol was administered at 0.05 μg/kg/day (typically 0.25–0.5 μg one to three times daily), whereas paricalcitol was given at higher doses at 0.15 mcg/Kg x 3/day (1–4 μg) (due to the relevant potency), with dose titration based on efficacy and safety. Cholecalciferol supplementation was provided to maintain adequate vitamin D status throughout follow-up. During treatment, all children with islet autoantibodies but without dysglycaemia or overt T1D became autoantibody-negative, with a median time to seroconversion of approximately seven months. Among children with pre-T1D or new-onset T1D, variable degrees of disease stabilization or remission were reported, and five patients who relapsed after treatment discontinuation became autoantibody-negative again after treatment was resumed. Treatment was generally well tolerated, with mild, reversible disturbances in mineral metabolism in eight patients (19%) requiring dose adjustment. Although these findings suggest that early VDR activation may help preserve immune tolerance and delay disease progression, the absence of a control group and the limited sample size warrant confirmation in larger randomized controlled trials.

Updated 15-year data from the PRECAL study, presented at ENDO 2026, included 103 children at high risk for T1D, with follow-up data available for 81 [54]. Using the same treatment protocol, T1Ab negativization was observed in 54 children within a median of 0.56 years, while seven who relapsed after treatment discontinuation negativized again after therapy was resumed. Of 10 children with T1D at presentation, six achieved complete remission for up to two years and one remained in remission for 2.9 years despite persistent positivity for four autoantibodies. Twelve children had not yet negativized and remained on paricalcitol at a median follow-up of 1.4 years. Hypercalciuria or hypercalcaemia occurred in 10 children, and one case of nephrocalcinosis resolved after dose adjustment or treatment discontinuation.

To the best of our knowledge, clinical evidence specifically evaluating the use of paricalcitol in T1D remains extremely limited. Apart from the PRECAL study, only one additional clinical study has investigated paricalcitol in patients with T1D. Jorgensen et al. conducted an RCT in 48 individuals with T1D and diabetic nephropathy, evaluating the effect of paricalcitol (1–2 μg/day) on urinary albumin secretion for 12 weeks. Paricalcitol reduced urinary albumin excretion rate by 18% compared with placebo; however, inflammatory or immunomodulatory markers were not assessed [55]. Thus, this study provides evidence for a renal effect of paricalcitol in T1D, but does not directly support an immunomodulatory effect.

## 5. Safety Profile and Future Perspectives

According to the Food and Drug Administration (FDA), paricalcitol is currently licensed for the prevention and treatment of SHPT in CKD stages 3–5D, with oral and intravenous dosing determined by CKD stage, baseline PTH concentrations and treatment response [55,56]. Dose adjustment requires monitoring of serum calcium, phosphate and PTH because excessive VDR activation may disturb calcium metabolism. Its use in pediatric populations is also approved for SHPT in specific age groups, whereas data during pregnancy remain limited [53,56,57].

Overall, paricalcitol was well tolerated in the available clinical studies. The most frequently reported adverse events were mild and transient disturbances in mineral metabolism, mainly hypercalcemia and hypercalciuria, which resolved after dose reduction or temporary treatment interruption. In the PRECAL study, mild hypercalcemia or hypercalciuria occurred in a minority of patients and was successfully managed by dose adjustment [53]. Similarly, transient hypercalcemia reported in the ophthalmological case normalized after reducing the paricalcitol dose [44]. Compared with calcitriol, paricalcitol appears to have lower calcemic activity, an important consideration if prolonged treatment is evaluated for immunomodulatory responses [3,44,57]. A recent prospective comparative study in hemodialysis patients reported a greater PTH-lowering effect with paricalcitol than with calcitriol, without clinically important hypercalcemia or hyperphosphatemia; the authors suggested that tighter PTH control may be beneficial in patients at risk of high-turnover renal osteodystrophy [58]. Apart from disturbances in mineral metabolism, paricalcitol has been associated with gastrointestinal adverse events, mainly nausea (12.9%) and diarrhea (11.5%). Dizziness, hypertension and edema have been reported less often [56].

At present, no dosing regimen has been established for the immunomodulatory use of paricalcitol, which remains off-label. Across available clinical studies, oral doses of 1–2 μg/day have been associated with changes in anti-inflammatory markers in patients with CKD and kidney transplant recipients, whereas PRECAL used considerably higher doses at 0.15 mcg/Kg × 3/day (1–4 μg) in children at high risk of T1D. It remains unknown whether immunomodulatory effects require different exposure from that used for SHPT. The optimal treatment regimen needs to be defined in dose-ranging studies.

The ability of paricalcitol to modify immune responses has also prompted interest in its potential role in cancer. In a preclinical study, Bandi et al. used human pancreatic cancer cell lines and an orthotopic murine model of pancreatic ductal adenocarcinoma to investigate the effects of paricalcitol in combination with hydroxychloroquine [59]. Animals were allocated to four treatment groups (vehicle, paricalcitol plus hydroxychloroquine, 5-fluorouracil plus oxaliplatin, and combination therapy). The combination significantly enhanced the antitumor effect of 5-fluorouracil and oxaliplatin, while promoting extracellular matrix remodeling and activation of NK, TNK and γδ T cells. Downregulation of integrin β4 (ITGB4) and extracellular matrix proteins suggested improved immune cell infiltration and chemosensitivity. However, as paricalcitol was administered together with hydroxychloroquine, its specific contribution to these immunomodulatory effects could not be determined. A single-arm phase II clinical trial subsequently enrolled 32 patients with resectable or borderline resectable/locally advanced pancreatic ductal adenocarcinoma, who received neoadjuvant nab-paclitaxel, cisplatin and gemcitabine together with intravenous paricalcitol (25 μg) administered on days 1 and 8 of each 21-day treatment cycle for up to six months [60]. CA19-9 normalization was achieved in 43.8% of patients and R0 resection in 59.4%, with an overall acceptable safety profile.

Clinical results in advanced pancreatic cancer remain limited and mixed. Chung et al. conducted a randomized, double-blind, placebo-controlled phase II trial in 24 patients with metastatic pancreatic ductal adenocarcinoma receiving pembrolizumab with either intravenous paricalcitol (25 μg three times weekly) or placebo [45]. No significant differences were observed in progression-free survival, overall survival, or treatment-related adverse events between the two groups. The authors proposed the short half-life of intravenous paricalcitol as one possible explanation for the lack of efficacy. Similarly, a phase II, open-label, single-arm trial evaluated oral paricalcitol (12 μg/day) in 15 patients with advanced pancreatic ductal adenocarcinoma. Paricalcitol was administered continuously in 28-day cycles until disease progression or unacceptable toxicity [46]. This study was terminated early after a planned interim analysis because the predefined efficacy threshold was not achieved, although the treatment was generally well tolerated. More recently, in a phase II RCT, 36 patients with previously untreated metastatic pancreatic ductal adenocarcinoma were assigned to gemcitabine and albumin-bound paclitaxel (GA) plus placebo, intravenous paricalcitol (75 μg weekly), or oral paricalcitol (16 μg daily) [47]. Paricalcitol was generally well tolerated, although grade 2–4 hypercalcemia occurred in 5 of 12 patients receiving oral paricalcitol and required dose reduction. In paired tumor biopsies, paricalcitol treatment was associated with reduced αSMA-positive fibroblasts and increased infiltration and spatial proximity of CD8+ T cells to tumor cells, suggesting modulation of both stromal and immune components of the tumor microenvironment. Partial responses were observed in 42% of patients in the combined paricalcitol arms compared with 9% in the placebo arm, although this study was not designed to assess comparative efficacy. Higher baseline tumor VDR expression was associated with partial response in the paricalcitol arms, supporting its potential role as a predictive biomarker [47].

Several clinical trials have attempted to evaluate paricalcitol beyond its approved indications, although progress has been limited. A phase III crossover trial in IgA nephropathy (NCT00599963) was terminated because of funding limitations, while two other trials—one in multiple myeloma (NCT00258258) and one in prostate cancer with bone metastases (NCT00634582)—were discontinued due to poor recruitment. Ongoing studies may provide further insights into potential applications outside CKD. The ALLIANCE trial (NCT05664880) is evaluating whether oral paricalcitol (2 μg/day) can improve symptoms, imaging findings and inflammatory markers in patients with chronic pancreatitis.

## 6. Discussion

Experimental studies have shown that paricalcitol affects several components of the immune response, including dendritic-cell function, T-cell activation, Treg/Th17 balance and inflammatory signaling. Evidence in humans, however, is much more limited and derives mainly from studies in patients with CKD or kidney transplantation. Some of these studies reported reductions in inflammatory markers, but the findings have not been consistent, particularly in randomized trials. Whether the immunomodulatory effects observed experimentally are clinically relevant, especially outside renal disease, remains unclear. Several mechanisms identified in experimental studies have not yet been demonstrated in humans. For example, paricalcitol suppressed renin gene expression by 30–50% in animal models of renal failure, but an RCT in patients with CKD found no significant effect on circulating renin or angiotensin II, suggesting that this effect may not translate to standard clinical doses [15,31]. Similarly, the effects of paricalcitol on pancreatic inflammation and β-cell function described in experimental diabetes have not been investigated in the clinical setting. Other potentially relevant mechanisms, including activation of the Nrf2/HO-1 pathway, modulation of SIRT1/SIRT3 signaling and the neuroprotective effects observed in experimental models, also remain limited to preclinical studies. These discrepancies underline the difficulty of extrapolating findings from experimental models to the clinical setting.

Among these emerging mechanisms, the modulation of granulomatous inflammation represents another area of growing interest. Granuloma macrophages express high levels of VDR, making them a potential target for paricalcitol [10]. In an experimental model of granulomatous inflammation, early administration of paricalcitol increased PD-L1 expression, promoted granuloma maturation, and reduced T-cell infiltration, consistent with a more regulated immune response. These findings raise the question of whether paricalcitol may have therapeutic value in diseases characterized by dysregulated granulomatous inflammation, including sarcoidosis. The ability of paricalcitol to upregulate PD-L1 expression across multiple cell types has provided the rationale for evaluating its combination with immune checkpoint inhibitors in pancreatic cancer. While results in early-stage disease are encouraging, studies in advanced or metastatic pancreatic cancer have failed to demonstrate relevant clinical benefit.

Paricalcitol is not without limitations as an immunomodulatory agent, and the evidence reviewed here raises more questions than it answers. Most clinical data derive from CKD and kidney transplant recipients, populations in which chronic inflammation, disturbances in vitamin D metabolism and underlying indication for VDR activation may influence treatment response. Whether the same effects are possible in individuals without CKD or in earlier stages of autoimmune disease remains unclear. Another important question is whether it is possible to identify patients good responders to paricalcitol. VDR polymorphisms, baseline 25-hydroxyvitamin D levels, circulating Treg/Th17 ratios, and inflammatory biomarkers such as CRP have been suggested as possible candidates, but none has been validated for patient selection.

The absence of RCTs in autoimmune endocrine conditions represents an important gap. Hashimoto thyroiditis, Graves’ disease and T1D involve immune pathways that overlap with those modulated by paricalcitol targets in preclinical models, including Treg expansion, DC tolerogenicity, and Th1/Th17 suppression. Whether modulation of these pathways can be translated into therapeutic benefit in endocrine autoimmunity remains unknown. The PRECAL study is the closest we have, and it was small, uncontrolled and used calcitriol alongside paricalcitol, making it hard to draw firm conclusions about paricalcitol specifically. Beyond endocrine conditions, paricalcitol has also been proposed as a candidate adjunct to biological therapies in rheumatoid arthritis, given its complementary immunomodulatory profile—particularly its capacity to suppress Th17 responses and expand Tregs—and its more favorable calcemic profile compared with calcitriol. Its effects have not, however, been tested in clinical trials. Taken together, the available evidence positions paricalcitol as a promising immunomodulatory agent with potential applications extending beyond CKD. However, its future clinical role will depend on well-designed mechanistic studies and adequately powered RCTs that establish efficacy, identify responsive patient populations, and define the diseases most likely to benefit from treatment. As far as T1D is concerned, oral paricalcitol has demonstrated promise in reducing T1D autoantibodies and mitigating disease progression across various stages, albeit with important acknowledged limitations [61].

## 7. Conclusions

Paricalcitol has a strong mechanistic rationale as an immunomodulatory VDR agonist with experimental evidence supporting effects on innate and adaptive immunity, oxidative stress and fibrosis. While clinical relevance warrants further research, paricalcitol is the only commercially available calcitriol analog approved for worldwide clinical use. Evidence in humans is largely restricted to CKD and kidney transplantation, while data on autoimmune endocrine disease still remain limited. Future studies with targeted dosing taking in regard to VDR potency and paricalcitol pharmacokinetics should evaluate whether clinically relevant immune modulation can be delivered, which patients are more likely to respond, and at which dosing.

## Figures and Tables

**Table 1:** Mechanisms of immunomodulatory action of paricalcitol.

Target	Effect	Immune Consequence
Dendritic cells (DCs) [9,10]	↓ MHC-II, ↓ CD80/CD83/CD86, ↓ IL-12; ↑ PD-L1; tolerogenic phenotype	↓ T cell priming → impaired initiation of autoimmune response
Endothelial adhesion molecule [23]	↓ ICAM-1, ↓ VCAM-1, ↓ fractalkine, ↓ TNF-α via ↓ NF-κB	↓ Leukocyte recruitment → ↓ myocardial inflammation; ↑ microvascular integrity
MAPK pathway [17,18]	↓ MAPK activation; ↓ macrophage infiltration; ↓ CD68; ↓ pro-inflammatory cytokines	↓ Innate immune-driven organ inflammation
Pancreatic β-cells/NLRP3 inflammasome [27]	↓ TNF-α, ↓ IL-1β via ↓ NLRP3; ↑ IL-2, ↑ adiponectin; ↑ SOD, ↑ CAT, ↑ GSH, ↑ GPx; ↓ MDA; ↑ fasting insulin, ↑ C-peptide; ↓ glucose, ↓ HOMA-IR	↓ Pancreatic inflammation and oxidative stress → preservation of islet architecture → improved β-cell function (T2DM rat model)
Granuloma macrophages [11]	↑ PD-L1 expression; ↓ CD68+ macrophages; ↓ CD3+ T cells; ↑ mature granuloma formation	↓ Excessive T cell activation → immune checkpoint modulation → granuloma stabilization
Regulatory T cells (Tregs) [12]	↑ CD8+ Foxp3+ Tregs (↑ CTLA-4, ↑ TGF-β); trend ↑ CD4+ Tregs → ↓ IL-17	↓ Th17-mediated inflammation → ↓ renal inflammation & fibrosis
NF-κB pathway [10,14]	VDR/NF-κB complex → ↓ promoter binding; ↓ NF-κB acetylation via SIRT1/SIRT3 → ↓ IL-6, ↓ TNF-α, ↓ IL-1β, ↓ IL-8	Suppression of chronic inflammation
SIRT1/SIRT3—NFκB/ FOXO3a [19]	↑ SIRT1/SIRT3 → ↓ NFκB acetylation → ↓ IL-1β, ↓ TNF-α; ↓ FOXO3a acetylation → ↑ MnSOD, ↑ catalase	↓ Hepatic inflammation and oxidative stress (NAFLD)
Nrf2/HO-1 pathway [15]	↑ Nrf2 nuclear translocation → ↑ HO-1, ↑ SOD, ↑ catalase, ↑ GSH; ↓ ROS, ↓ MDA	↓ Oxidative stress → ↓ inflammation-driven tissue injury
TGF-β/Smad pathway [17]	↓ TGF-β1/Smad2/3 activity; ↓ mesenchymal markers (α-SMA, vimentin, collagen I)	↓ Epithelial-to-mesenchymal transition → ↓ renal fibrosis
RAAS/Renin [16]	↓ Renin, ↓ angiotensinogen, ↓ renin receptor mRNA	↓ Angiotensin II → ↓ NF-κB activation → indirect anti-inflammatory effect
Iron metabolism/GSK3β [21]	↓ IRP2 → ↓TFR → ↓ brain iron accumulation → ↓ GSK3β (Tyr216) → ↓ Tau phosphorylation (Thr181, Ser396)	↓ Tau pathology → neuroprotection in Alzheimer’s disease

Abbreviations: DC, dendritic cell; Treg, regulatory T cell; MHC-II, major histocompatibility complex class II; IL, interleukin; TNF-α, tumour necrosis factor-alpha; TGF-β, transforming growth factor-beta; NF-κB, nuclear factor kappa-light-chain-enhancer of activated B cells; VDR, vitamin D receptor; SIRT, sirtuin; FOXO3a, forkhead box O3a; RAAS, renin–angiotensin–aldosterone system; Nrf2, nuclear factor erythroid 2-related factor 2; HO-1, heme oxygenase-1; ROS, reactive oxygen species; SOD, superoxide dismutase; GSH, glutathione; MDA, malondialdehyde; MnSOD, manganese superoxide dismutase; GPx, glutathione peroxidase; CAT, catalase; MAPK, mitogen-activated protein kinase; α-SMA, alpha-smooth muscle actin; NAFLD, non-alcoholic fatty liver disease; PD-L1, programmed death-ligand 1; CTLA-4, cytotoxic T-lymphocyte-associated protein 4; ICAM-1, intercellular adhesion molecule-1; VCAM-1, vascular cell adhesion molecule-1; IRP2, iron regulatory protein 2; TFR, transferrin receptor; GSK3β, glycogen synthase kinase 3 beta; NLRP3, NOD-like receptor protein 3; HOMA-IR, homeostasis model assessment of insulin resistance; T2DM, type 2 diabetes mellitus; TXNIP, thioredoxin-interacting protein; ↑, increase or upregulation; ↓, decrease or downregulation.

**Table 2:** Clinical studies and case reports evaluating clinical and immunomodulatory effects of paricalcitol.

Author (Year)	Disease	Study Type	Population	Dose	Duration	Immune Outcomes	Clinical Outcomes
Lucisano et al. (2015) [28]	CKD/SHPT	Prospective observational	40 CKD stage 4–5 + SHPT; 20 controls	IV 5 μg single dose	24 h	↓ IL-17, IL-6, IL-1β, TNF-α, IFN-γ, NGAL, ESR (*p* < 0.0001); confirmed in stimulated PBMCs	↓ ESR; hsCRP, albuminuria, PTH: non-significant trend
Izquierdo et al. (2012) [29]	CKD/HD/SHPT	Prospective observational	19	IV 7.5 or 15 μg/week (dose-adjusted per mineral metabolism)	12 weeks	↓ CRP, TNF-α, IL-6, IL-18; ↑ IL-10; ↓ MDA, ↓ nitrites, ↓ protein carbonyl groups; ↑ SOD, ↑ catalase, ↑ GSH, ↑ thioredoxin	↓ Inflammatory burden; ↓ oxidative stress
Alborzi et al. (2008) [30]	CKD stage 2–3	RCT	24	Oral placebo, 1 or 2 μg/day (3 arms)	4 weeks	N/A	↓ hsCRP (2 μg/day only); ↓ albuminuria (both doses); no change in BP, GFR
Lundwall et al. (2015) [31]	CKD stage 3–4	RCT	36	Oral placebo, 1 or 2 μg/day (3 arms)	12 weeks	N/A	Preservation of endothelial function (FMD) in 2 μg/day arm; no change in BP, PWV, albuminuria, CRP
Larsen et al. (2013) [32]	CKD stage 3–4 + albuminuria	RCT	26 C	Oral 2 μg/day vs. placebo	6 weeks	N/A	↓ Albumin excretion under experimental conditions; no change in renin, AngII, aldosterone
Riccio et al. (2015) [33]	CKD stage 3b–5 + anaemia	RCT	60	Oral 1 μg/day vs. calcitriol	24 weeks	N/A	↑ Haemoglobin; ↓ proteinuria; hsCRP: unchanged
Pihlstrøm et al. (2020) [34]	Kidney transplant (de novo)	RCT	74	Oral 2 μg/day vs. standard care	40 weeks	↑ OPG (+21% TNFRSF11B gene expression in biopsy); no change in 13 other biomarkers	N/A
Donate-Correa et al. (2017) [35]	Kidney transplant + SHPT	Prospective observational	31	Oral 1 μg/day	12 weeks	↓ IL-6 (29%), ↓ TNF-α (9.5%); ↓ TNF-α/IL-10 and IL-6/ IL-10 ratios; IL-10: non-significant trend ↑	↓ hsCRP; ↓ PTH
Oblak et al. (2017) [36]	Kidney transplant + proteinuria	RCT	168	Oral 2 μg/day vs. placebo	24 weeks	↓IL-6 (29%); ↓TGF-β (12%)	Anti-inflammatory and anti-fibrotic effect; renin and aldosterone: unchanged
Uriol-Rivera et al. (2024) [37]	IgA nephropathy (refractory)	Prospective interventional	7	Oral 1 μg/day → + secukinumab	112 weeks	↓ Th2 cells; ↓ Th2/Treg ratio; ↑ Th1/Th2 ratio; Th1, Th17, Treg: unchanged	↓ Proteinuria 71%; ↓ eGFR decline
Thethi et al. (2015) [39]	T2DM + CKD stage 3–4	RCT	60	Oral 1 μg/day vs. placebo	12 weeks	N/A	No significant endpoints due to low dose of paracalitol (endothelial function, hsCRP, IL-6, TNF-α)
de Zeeuw et al. (2010) [40]	T2DM + albuminuria	RCT	281	Oral placebo, 1 or 2 μg/day (3 arms)	24 weeks	N/A	↓ Albuminuria (2 μg/day)
Durakovic et al. (2004) [41]	Psoriasis (plaque)	RCT	11	Topical 15 μg/g once daily	12 weeks	N/A	↓ Erythema, scaling, plaque thickness vs. placebo; no change in calcium metabolism
Bothou et al. (2018) [42]	Atopic dermatitis (severe)	Prospective case report	1 paediatric patient	Oral 2 μg × 3/day	3 years	N/A	Disease remission maintained; ↓ urinary calcium vs. calcitriol
Papadimitriou et al. (2021) [43]	Alopecia universalis	Prospective case series	1 paediatric patient	Oral 2 μg × 3/day	24 weeks	N/A	Partial hair regrowth (scalp, eyebrows, eyelashes); relapse after discontinuation of paricalcitol
Nemet et al. (2024) [44]	HSV-1 keratitis + corneal perforation	Prospective case report	1 patient	Oral 1 μg every other day → 1 μg twice weekly	~36 weeks	N/A	Corneal re-epithelialisation; no HSV recurrence; transient ↑ serum Ca → dose reduction
Chung et al. (2025) [45]	Metastatic PDAC	RCT	24	IV 25 μg 3×/week + PEM vs. placebo + PEM	Variable; Until progression/toxicity	N/A	No significant differences in PFS, OS, or treatment-related AEs
Easty et al. (2026) [46]	Advanced PDAC	Open-label, single-arm	15	Oral 12 μg/day + gemcitabine/nab-paclitaxel	1–36 weeks	N/A	Trial terminated early after interim analysis; predefined efficacy threshold not met; generally well tolerated
Perez et al. (2026) [47]	Metastatic PDAC	RCT	36	IV 75 μg/week or oral 16 μg/day + GA vs. placebo + GA	Variable; >52 weeks in long-term responders	↓ αSMA+ fibroblasts; ↑ total T-cell and CD8+ T-cell density; ↑ CD8+ T-cell proximity to tumour cells	PR: 42% vs. 9% with placebo; grade 2–4 hypercalcaemia in 5/12 oral arm; higher baseline tumour VDR associated with PR

Abbreviations: CKD, chronic kidney disease; SHPT, secondary hyperparathyroidism; HD, haemodialysis; RTR, renal transplant recipient; T2DM, type 2 diabetes mellitus; r-IgAN, refractory IgA nephropathy; HSV, herpes simplex virus; RCT, randomised controlled trial; IL, interleukin; TNF-α, tumour necrosis factor-alpha; IFN-γ, interferon-gamma; TGF-β, transforming growth factor-beta; NGAL, neutrophil gelatinase-associated lipocalin; ESR, erythrocyte sedimentation rate; CRP, C-reactive protein; hsCRP, high-sensitivity CRP; OPG, osteoprotegerin; FMD, flow-mediated dilation; NMD, nitroglycerine-mediated dilation; PWV, pulse wave velocity; BP, blood pressure; GFR, glomerular filtration rate; AngII, angiotensin II; MDA, malondialdehyde; SOD, superoxide dismutase; GSH, glutathione; PBMCs, peripheral blood mononuclear cells; N/A, not assessed/not applicable. PDAC, pancreatic ductal adenocarcinoma; PEM, pembrolizumab; PFS, progression-free survival; OS, overall survival; AEs, adverse events; GA, gemcitabine and albumin-bound paclitaxel; αSMA, alpha-smooth muscle actin; VDR, vitamin D receptor; PR, partial response; ↑, increase or upregulation; ↓, decrease or downregulation.

## Data Availability

No new data were created or analyzed in this study. Data sharing is not applicable to this article.
